# Square wave voltammetry based electrochemical determination of affinity of cholesterol triethylene glycol modified DNA-aptamers for protoporphyrin IX

**DOI:** 10.1016/j.heliyon.2023.e18861

**Published:** 2023-08-05

**Authors:** Abdul Wahab Aliyu, Muhammad Najmi Mohd Nazri, Nur Fatihah Mohd Zaidi, Khairul Mohd Fadzli Mustaffa

**Affiliations:** a Institute for Research in Molecular Medicine, Health Campus, Universiti Sains Malaysia, 16150 Kubang Kerian, Kota Bharu, Kelantan, Malaysia; b Pharmaceutical Microbiology and Biotechnology, Faculty of Pharmaceutical Sciences, Gombe State University, P.M.B. 127, Tudun Wada, Gombe State, Nigeria

**Keywords:** Protoporphyrin IX, Aptamers, Cholesterol, Modification, Affinity

## Abstract

Recent advancement in molecular medicine has seen applications of advanced biotechnology tools such as aptamer technology in therapeutics and diagnostics. Aptamer technology has witnessed various approaches including “Click-Chemistry” towards modifying aptamer structure to improve its potentials, but limited studies have reported the influence of such alteration on aptamer's specificity and affinity for their targets. Here, we utilized square wave voltammetry (SWV) electrochemical sensing based on heme to show the effects of cholesterol-triethylene-glycol (COL-TEG) modification of protoporphyrin-IX DNA-aptamers (OKA_24 and OKA_26) on their affinity for heme. Binding was evaluated by immobilizing 5 μM of heme onto cysteamine-glutaraldehyde-coated gold-electrode to construct electrochemical biosensor. Sensing of native/modified-aptamer was achieved by incubating their varying concentrations (9.76 nM - 10 μM) with heme-coated gold-electrode in HKSCM buffer pH 5, for 15 min. Chloroquine (2.5 μM) and non-binding HPIX-aptamer (2.5 μM) served as controls. Ferrocene was the redox solution used for SWV analysis. Protoporphyrin-IX DNA-aptamers specificity for heme was not tarnish by lipid conjugation. Selective binding of 2.5 μM of COL-TEG-OKA_24 and COL-TEG-OKA_26 to heme induced peak-current reduction by 30.68% and 24% respectively. Incubation of OKA_24 and OKA_26 aptamers produced resistance to current flow through the heme-coated gold-electrode by 23.21% and 14.4 8% respectively. Affinity SWV reveals that cholesterol conjugation decreases the affinity of COL-TEG-OKA_24 ($K_{D}$ = 4 7.13 ± 3.767 nM) and COL-TEG-OKA_24 ($K_{D}$ = 84.6 ± 8.7 nM) by 3- fold. There is a need to check the impact of such alteration on inhibition of heme to hemozoin polymerization, a process mediated by *Plasmodium falciparum*.

## Highlights

• Protoporphyrin IX-coated gold electrode for binding of Heme DNA-aptamers was fabricated.
• Square wave voltammetry of cholesterol-based heme DNA-aptamer bioconjugate was investigated.
• Bioconjugation of cholesterol with heme DNA-aptamer does not tarnish binding properties.

## 1 Introduction

Biological macromolecules are known for their vital role in the maintenance of the structure and function of a normal cell. However, recent advancements in the field of molecular medicine have allowed their application in therapy and diagnosis of abnormality in cell, tissue, organ and mammalian system [1], [2]. Among the four major macromolecules, Nucleic acid has received wider applications especially in the form of aptamers [3]. Aptamers are small length nucleotide sequence of single strand RNA or DNA with desirable biological activities such as specific molecular target recognition. They are also antigenic-free biologics that can be selected to form a complex with molecular target of interest due to their ability to assume three-dimensional conformation [4], [5], [6]. Numerous aptamer target molecules have been reported. A good example is the chloroquine molecular target (Heme), a byproduct of hemoglobin degradation mediated by *Plasmodium falciparum* (*P. falciparum*) during parasite intra-erythrocytic growth and development [7], [8]. Heme is toxic to *P. falciparum*, but the parasite evades this toxicity by converting heme to non toxic hemozoin [9]. Heme DNA-aptamers (OKA_24 and OKA_26) with profound affinity for their target have been in vitro selected [10]. Although heme binding aptamers have demonstrated good in vitro inhibition of hemozoin production, their potential therapeutic application is limited by their lack of ability to permeate red blood cell (RBC) membrane to reach the parasite food vacuole [10]; therefore molecular modification of heme binding aptamer is required to confer them with such desirable properties. Modification in the structure of aptamer (pentose sugar, phosphodiester bond and 3' or 5' terminal ends) is often employed to incorporate the later with good biological capabilities including, serum stability, cell permeability and or improved efficacy [11], [12], [13]. The most widely used structural modification approach in drug discovery and development is “click chemistry” which involves combining desirable properties of two or more molecules into single agent via chemical synthesis. [14], [11]. However limited study reported the influence of such alteration on the inherent properties of aptamer such as specificity and affinity for their target. In this work, we used square wave voltammetric electrochemical sensing based on heme to demonstrate the impacts of cholesterol triethylene glycol modification of heme-DNA aptamers on the specificity and affinity for their target.

## 2 Materials and methods

### 2.1 Chemicals and reagents

Cholesterol triethylene glycol-heme aptamers were synthesized and supplied by GENEWIZ, China. Diamond and polishing pads, 0.05 μM polishing alumina slurry and 1.0 μM polishing diamond slurry were obtained from ALS, BAS Inc., Japan. 3 mm gold electrode (Gold voltammetry (Au) MF-2014, BASi). All chemicals and solutions used were of analytical grade. 0.1M H_2_SO_4_ was obtained from Amresco, USA. 5 mM HCl and 1.5 mM MgCl_2_ Merck, USA. Cysteamine (1 mM) and 2.5% glutaraldehyde Sigma Aldrich, USA. One milli molar (1M) Tris pH 7.4 (Bio-Rad) Malaysia. Nova 2 Software version, Voltammeter (μAUTOLAB Type III). Protoporphyrin IX (Heme) was obtained from MedChemExpress LLC, USA. Nuclease-free water and Dimethyl sulfoxide (DMSO) from Apical Scientific, Malaysia.

### 2.2 Preparation of aptamer incubation buffer

The buffer system used to evaluate the biding of aptamer/conjugate for heme consists of 20 mM HEPES (Sigma Aldrich, USA, H3376-500g), 5 mM potassium chloride (Sigma Aldrich, USA), 120 mM sodium chloride (Biotechnology grade, 1 kg, first Base), 1 mM calcium chloride (Sigma Aldrich, USA, C5080-500G) and 1 mM magnesium chloride Sigma Aldrich, USA (M8266-100g), (HKSCM) buffer pH 5. The HKSCM buffer was formed by weighing 4780 mg of HEPES, 372.5 mg of potassium chloride, 700 mg of sodium chloride, 110.9 mg of calcium chloride and 203 mg of magnesium chloride. The solutes were placed in a calibrated Scott bottle holding about 750 ml of double-distilled water. To dissolve and generate a clear solution, the mixture was repeatedly stirred. 3M HCl was used to adjust the pH to 5 and doubled distilled water (ddH_2_O) was transferred into the Scott bottle to 1 L mark. Suction filtration using 0.22 m*μ* nylon membrane was used to sterilize the buffer. The HKSCM buffer was kept at 4 ^∘^C until needed.

### 2.3 Phosphate buffered saline tween 20 (washing buffer)

The Phosphate buffered saline tween 20 (PBST) containing 1.8 mM potassium phosphate, 10 mM sodium phosphate, 137 mM sodium chloride and 2.7 mM potassium chloride (1X PBS) and 0.05% tween 20 was prepared by dissolving 8 g of sodium chloride, 2.7 g of potassium chloride, 1.42 g of sodium phosphate and 0.24 g of potassium phosphate in 800 ml of double distilled water, the pH was measured and then adjusted to 7.4 using 3M HCl. The solution was transferred to a calibrated Scott bottle, and double distilled water was added to the 1000 ml mark. Finally, the 250 μl of solution was removed and replaced with an equivalent amount of tween 20. The PBST was kept at 4 ^∘^C until required.

### 2.4 Preparation of standard (STD) stock solution of heme binding DNA-aptamers

To the tube containing lipolyzed OKA_24, OKA_26, COL-TEG-OKA_24, COL-TEG-OKA_26 and HPIX-nonbinding (control aptamer), 169 μl, 189 μl, 184 μl and 136 μl of nuclease-free water was respectively added to make 100 μmol/L/tube stock solutions. Reconstituted unmodified/modified aptamer solutions were mixed thoroughly by spinning down for 45 sec and kept at −20 ^∘^C to be thawed when required.

### 2.5 Preparation of working STD solution of heme

From the 1 mL (100 μM) heme stock profile, a 5 μM working heme solution was made by pipetting 5 μl of the stock in to a 1 ml tube containing 95 μl of DMSO in the dark. The resulting working solution was then mixed by gently pipetting up and down. The tube was then covered with aluminum foil and kept at −20 ^∘^C until needed.

### 2.6 Preparation of aptamer working concentration

The HKSCM incubation buffer pH 5 prepared as above was used to make modified/unmodified aptamer working concentration from the STD modified/unmodified aptamer stock solutions. The 10 μM working concentration of the ligand was prepared by pipetting 10 μl of 100 μM stock solution into 90 μl of HKSCM incubation buffer. The resulting 100 μl of 10 μM working modified/unmodified aptamer concentration was serially diluted by pipetting 15 μl into 1 ml falcon tube containing 15 μl HKSCM buffer. The solution was mixed by upward and downward Pipetting. Subsequent concentrations were prepared by removal of 15 μl from previous concentration to a tube containing 15 μl of buffer alone followed by mixing as above.

### 2.7 Polishing and cleaning of the electrode

To polish the 3 mm gold electrode, 2 drops of 1.0 μM polishing diamond slurry (ALS, BAS Inc., Japan) was dropped on the diamond polishing pad (ALS, BAS Inc., Japan). The gold surface of the electrode was then placed perpendicularly onto the slurry and gently moved against the diamond pad in an 8-shaped direction for about 2 min. The electrode was then washed with ddH_2_O followed by 70% ethanol. The same procedure was repeated with 0.05 μM polishing alumina slurry (ALS, BAS Inc., Japan) on a separate aluminum polishing pad. The electrode was again washed with ddH_2_O and then with 70% ethanol. The electrode was then sonicated for 10 min in 70% EtOH to remove the excess slurry. Next the electrode was immersed in piranha solution for 2 minutes, washed with ddH_2_O, followed by 70% EtOH. Finally, the gold electrode was cleaned electrochemically using the cyclic voltammetry (CV) technique for three repeated scans in 0.1M H_2_SO_4_. Washing was repeated as above and SWV on the bare electrode was recorded to get a baseline reading.

### 2.8 Immobilization of heme onto gold electrode (biomolecular recognition elements)

Fabrication of heme biosensor was achieved via immobilization of heme onto gold electrode for biosensing of heme-binding DNA-aptamers. The previously polished and cleaned electrode was incubated with newly made 1 mM cysteamine for 2 hours. Excess cysteamine was then removed by washing the electrode three times (3X) with PBST. The washed cysteamine-coated electrode was then incubated with freshly prepared 2.5% glutaraldehyde for 30 minutes. Unbound glutaraldehyde was removed by washing with PBST 3X as above. SWV was then performed and the peak current for gold-cysteamine-glutaraldehyde (Au-Cyst-Glu) was recorded. The electrode was then washed 3X with PBST prior to incubation with 5 μM heme for 1 hr. Incubation with heme was achieved by pipetting 5 μl of the heme directly onto the inverted gold-cysteamine-glutaraldehyde surface. The heme immobilized gold electrode was then washed 3X with PBST to remove excess unbound heme. Electrode immobilized with heme was stored in PBS at 4 ^∘^C until when required.

### 2.9 Specificity square wave voltammetric analysis

Heme binding and biorecognition of modified/unmodified aptamers was carried out by subjecting the heme-coated gold electrode to square wave voltammetric analysis using ferrocene as a redox medium. Incubation of modified/unmodified was done in HKSCM buffer by pipetting 10 μl of 2.5 μM of modified/unmodified aptamers, chloroquine and control non-binding HPIX aptamer, directly onto the heme-coated gold surface of the inverted electrode for 15 min. The electrode was then washed 3X with PBST to remove unbound ligand. Square wave voltammetric analysis was then performed and the change in peak current representing formation of the heme-aptamer complex was recorded.

### 2.10 Affinity square wave voltammetry of unmodified/modified aptamer with heme

**Figure 1:**
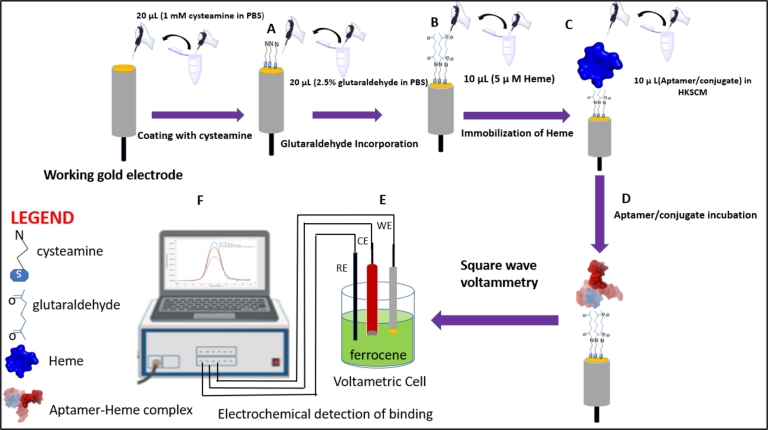
Schematic diagram illustrating the construction steps and working of a heme-based electrochemical sensor for determining the affinity of unmodified/modified aptamers. Coating gold surface with cysteamine (A); incorporation of glutaraldehyde on to cysteamine coated gold surface (B); immobilization of heme onto cysteamine-glutaraldehyde coated gold electrode (C); incubation of heme coted gold surface with aptamer/conjugate (D) and square wave voltammetry electrochemical analysis of binding interaction between heme and unmodified/modified aptamer (E).

**Figure 2:**
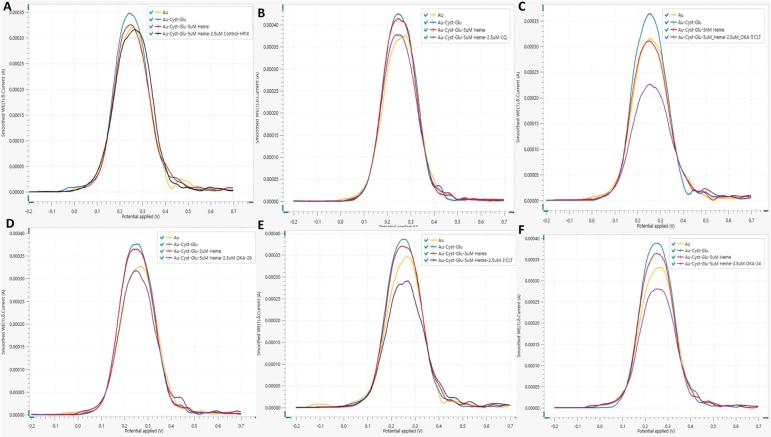
Voltammogram showing the heme binding interaction with Control-non-binding HPIX aptamer (A), Chloroquine (B) COL-TEG-OKA_26 (C), OKA26 (D), COL-TEG-OKA_24 (E) and OKA24 (F).

Square wave voltammetric affinity measurement of OKA_24, OKA_26, COL-TEG-OKA_24 and COL-TEG-OKA_26 for heme was performed in ferrocene using Voltammeter (μAUTOLAB Type III) as illustrated in Fig. 1. Redox analysis was conducted in 10 mL of ferrocene at 37 ^∘^C. The 5 μM working heme molecule was immobilized onto the cysteamine-glutaraldehyde coated gold electrode as described above. Two-fold serially diluted concentration of unmodified/modified aptamer (9.76 nM - 10 μM) were analyzed for complex formation with heme represented as peak current alteration as described in section 2.9 above. The working electrode was washed 3x with PBST before and after performing individual SWV for each concentration of unmodified/modified aptamer. The experiment was repeated three times and the average peak current for each concentration was measured. A plot of peak current and voltage potential was used to construct a voltammogram (Fig. 2).

### 2.11 Measurement of binding affinity ($K_{D}$ value)

To measure the binding affinity of modified/unmodified aptamers for heme, the change in current flow in the working gold electrode due to binding of the ligand (aptamer) with the immobilized heme moiety was read from the voltammogram until saturation was achieved. Voltammetric waves were recorded three times and the plot of change in peak current reduction (*δ*I/*μ*A) against analyte concentration [aptamer] was constructed to generate a saturation binding curve. Table 1 gives the summary of the optimal square wave voltammetry parameters whereas the measure of affinity ($K_{D}$ value) was determined from the binding curve as mathematically expressed below:

(1) Aptamer+Heme=HemeAptamer

At the point of saturation, the K equilibrium ($K_{eq}$) for heme binding can be expressed as

(2) $$K_{eq}=\frac{\left[HemeAptamer\right]}{\left[Heme][Aptamer\right]}=\frac{K_{on}}{K_{off}}=K_{A}$$

$K_{eq}$ = equilibrium constant, [Aptamer] = Aptamer molar concentration, [Heme] = Heme molar concentration and [HemeAptamer]= HemeAptamer complex molar concentration. $K_{A}$ = is constant for association at equilibrium and is mathematically represented by $K_{D}$ inverse, therefore,

(3) $$K_{A}=\frac{1}{K_{D}}=\frac{\left[HemeAptamer\right]}{\left[Heme][Aptamer\right]}$$

(4) $$K_{D}=\frac{\left[Heme][Aptamer\right]}{\left[HemeAptamer\right]}$$

**Table 1:** Optimal square wave voltammetry parameters.

Parameter	Value for method
Electrolyte	5 mM of K3[Fe(CN)6]
Cell temperature	37 ^∘^C
Working electrode type	Gold voltammetry (Au) MF-2014, BASi
Working electrode dimension	3 mm
On electrode Lord	10 μl
Incubation time	15 min

$K_{D}$ = is the concentration of Aptamer at saturation limit where [Heme] = [HemeAptamer]. The lower the $K_{D}$ value the higher the affinity and vice versa.

The fractional amount of HemeAptamer complex formed at equilibrium is the fractional saturation represented by *θ*

(5) $$\theta =\frac{\left[HemeAptamer\right]}{\left[Heme]+[HemeAptamer\right]}=\frac{K_{A}[Aptamer]}{1+K_{A}[Aptamer]}=\frac{\left[Aptamer\right]}{\left(K_{D}+[Aptamer]\right)}$$

[HemeAptamer] was obtained by calculating the value of change in peak current reduction of heme coated gold electrode due to the binding of aptamer while [Heme] is the initial peak current produced by immobilization of heme onto glutaraldehyde-coated gold electrode.

### 2.12 Statistical analysis

Experiments were carried out three times and data were analyzed using GraphPad prism software (version 7). Mean standard deviation was utilized to denote experimental parameters. Student t test was used to compare the mean $K_{D}$ of modified and unmodified aptamer, p value was set at < 0.05

## 3 Results and discussions

### 3.1 Modification of heme binding DNA-aptamer

A ligand's biological action is determined by its affinity for its molecular target. The interaction between ligand and target must occur in close proximity. To achieve a required proximity in vitro or in vivo, ligands sometimes need to travel in serum and cross through lipid bilayer, or nuclear membrane depending on the case [15]. Aptamer is not stable in serum due to exonuclease hydrolysis, and some cannot pass the lipid bilayer, hence its effectiveness is frequently impaired. Thanks to the “click chemistry” technique for allowing aptamers structure to be conjugated with appropriate compounds to impart stability or boost aptamer cell penetrating capability [16], [17], [7]. In this study, cholesterol triethylene glycol, a cell penetrating lipid, was conjugated to heme binding aptamer at 3' terminal end (Table 2) and then subjected to square wave voltammetry electrochemical measurement based on heme to assess the effects of this modification on the aptamer specificity and affinity for heme.

**Table 2:** Two dimensional (2D) structure of OKA_24 and OKA_26 aptamers and their cholesterol triethylene glycol aptamer conjugates.

2D structure of aptamer	Cholesteroltriethyleneglycol aptamer conjugates

### 3.2 Lipid modified aptamer retains heme binding property

**Figure 3:**
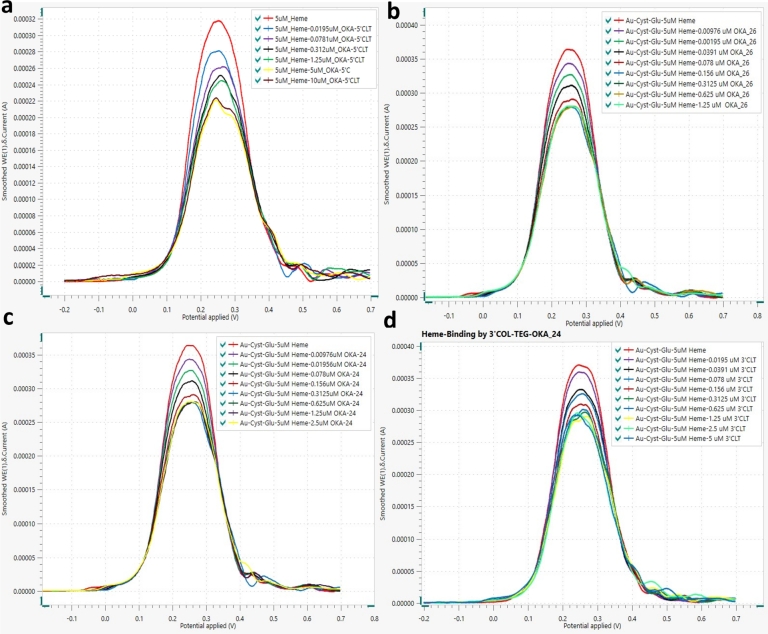
Voltammogram showing the heme binding by COL-TEG-OKA_26 (a), OKA_26 (b), OKA_24 (c) and COL-TEG-OKA_24 (d).

Chloroquine (CQ), a 4-aminoquinolines antimalarial agent, was the first line drug of choice in arresting blood stage malaria infection because of its safety and efficacy. CQ acts by binding to heme (a toxic by-product of hemoglobin degradation carried out by malaria parasite) and prevent it polymerization into parasite friendly hemozoin. Accumulation of heme in malarial parasite's digestive food vacuole induces toxicity that eventually kills the plasmodium [8], [9], [18]. This heme binding property of CQ has been found to be mimicked by OKA_24 and OKA_26 aptamers according to the finding of Niles and his team [10]. However, these aptamers lack the ability to enter red blood cell (RBC) and exert these effects, hence the need for their structural modification. Structural modification may modulate heme specificity and binding affinity of these oligonucleotides, therefore, a need aroused for investigating the effect of conjugating lipid (Cholesterol triethylene glycol) to OKA_24 and OKA_26 on their heme binding property. For justification of such concept, the specificity and affinity of lipid modified aptamer for heme was assessed using square wave voltammetry. The coating of a gold electrode with cysteamine and glutaraldehyde yielded a very high peak current. In quantitative and analytical terms, coating of gold electrode with cysteamine and glutaraldehyde produced a peak current (15.2%) higher than the base line peak current of bare gold electrode. The increase in the peak current with the changing potential was due to increase electron transfer to gold electrode surface as a result of increase surface area mediated by cysteamine-glutaraldehyde adsorption coupled with electrostatic interaction between the aldehyde terminals of Glu/Cyst/Au working electrode and the $[Fe(CN)6]3^{-}[Fe(CN)6]4^{-}$ couple. Contrary to the former, immobilization of heme reduces current conductance through cysteamine-glutaraldehyde coated working gold electrode by creating a barrier that decrease electron transfer to the working electrode producing lesser peak current compared to the former. A similar pattern was obtained by Liv [19] following incubation of S-gene on GluAl/CysAm/Au/ electrode for immuno-sensing the SARS-CoV-2 spike antibody. Electrochemical biosensing of 2.5 μM COL-TEG-OKA_24 and COL-TEG-OKA_26 in HKSCM buffer pH 5 demonstrate the effects of modification of the concerned ligands on specificity for heme compared to unmodified (OKA_24/OKA_26) and the controls (CQ and non-binding HPIX aptamer) (Fig. 2). Incubation of heme-coated gold working electrode with modified/unmodified aptamer produced profound peak current reduction by increasing the thickness of the electron limiting barrier. The peak current reduction was highest in modified conjugate; COL-TEG-OKA_26 (30.68%) and COL-TEG-OKA_24 (24%) moderate in unmodified; OKA_24 (23.21%), OKA_26 (14.48%) and CQ (8.15%) indicating profound specific affinity for heme. On the other hand, there was minimal or no change in peak current when non-binding HPIX aptamer was incubated with heme coated working gold electrode signifying the absence of evident binding and affinity for heme. These square wave results have shown that, conjugating cholesterol triethylene glycol to OKA_24 and OKA_26 does not alter their specificity for heme. This finding is in agreement with the report of Niles [10] on the influence of caping of these aptamers with inverted deoxythymidine. Similarly, spectral titration of heme with modified OKA_24 and OKA_26 aptamers has shown that such modification has not affect their specificity for heme [5]. Although the electrochemical properties dissipated by aptamer-conjugate have demonstrated that, lipid moiety conjugation to OKA_24 and OKA_26 aptamers, does not inhibit their heme binding properties, the need to get a quantitative measurement of the extent to which they have an affinity for heme was necessary. Therefore, affinity square wave voltammetry was carried out to determine $K_{D}$ value of the conjugated aptamer as described above. SWV was carried out using varying concentration of modified/unmodified aptamer (9.76 nM to 10 μM) as described in section 2.10 above; until saturation was attained (Fig. 3).

**Figure 4:**
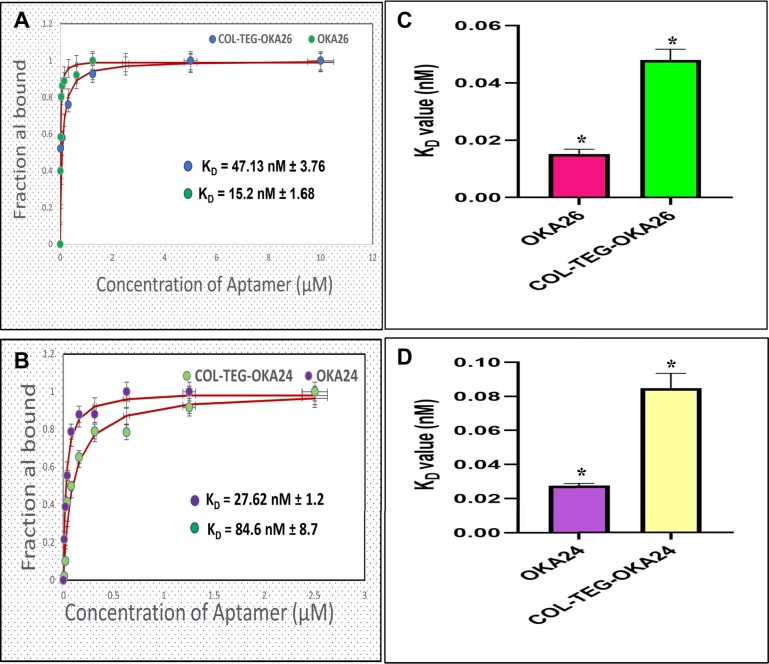
Saturation binding curve for modified/unmodified heme binding aptamers (A-B) and their corresponding affinity for heme (C-D).

The data from voltammogram (Fig. 3) was used to construct a saturation binding curve (Fig. 4) from which the $K_{D}$ was determined. It can be extracted from this experiment that conjugation of cholesterol triethylene glycol to OKA_24 ($K_{D}$ = 27.62 ± 1.2 nM) and OKA_26 ($K_{D}$ = 15.2 ± 1.68 nM) leads to decrease in binding affinity for heme by 3-fold in both COL-TEG_26-OKA ($K_{D}$ = 4 7.13 ± 3.767 nM) and COL-TEG_24-OKA ($K_{D}$ = 84.6 ± 8.7 nM) conjugate. Despite the fact that these findings disagreed with the prior findings obtained by carrying out an ultraviolet visible spectrum titrations of native and lipid conjugated aptamers with heme, [5], it is not surprising because of the role played by the environmental influence on the conformation of molecules which can either enhance or reduce their activity [20], [21]. Immobilization of heme on a glutaraldehyde-cysteamine coated gold electrode, for example, improved the binding affinity of both modified and unmodified aptamers for heme relative to their respective binding affinity in buffer solutions analyzed by UV spectral titration [5] (data not shown). However, this increase in binding favors unmodified aptamers with 3-fold increase compared to lipid conjugated once. It is important to note that immobilization of the heme moiety on working gold electrode provides a similar analogy to stationary stage of receptor in receptor-ligand interaction. These could explain the reasons behind obtaining the low $K_{D}$ measure for both modified aptamers, unlike the case with UV spectroscopy in which $K_{D}$ values were higher. Table 3 shows the respective measure of affinity of modified/unmodified aptamers.

**Table 3:** Affinity measure of cholesterol triethylene glycol modified and unmodified heme binding DNA-aptamers.

Aptamer/conjugate	*K*_*D*_ (nM)
COL-TEG-OKA_26	47.13 ± 3.767
OKA_26	15.2 ± 1.68
COL-TEG-OKA_24	84.6 ± 8.7
OKA_24	27.62 ± 1.68

## 4 Conclusion

The impact of cholesterol triethylene glycol conjugation to OKA_26 and OKA_24 DNA aptamers on their affinity for heme was established. Heme when immobilized onto a gold electrode binds to lipid modified protoporphyrin IX DNA aptamer 3-fold lesser than their unmodified counterparts. Despite the decrease in affinity for heme exhibited by OKA_26 and OKA_24 DNA aptamer because of modification with the lipid, the intended use of these aptamer (targeting erythrocytic stage of *Plasmodium falciparum*) made the need for lipid modification necessary. Cholesterol as a good cell penetrating lipid could, confer heme binding DNA aptamers with RBC penetration power which both OKA_26 and OKA_24 DNA are lacking. Although the pH of the buffer used in this study was within the pH range of the *Plasmodium falciparum* digestive vacuole, the site where the binding of antimalarial drugs and heme occurs; the findings cannot be directly extrapolated to in vivo rat models or human subjects. This is because, the use of non-physiological buffer limits the ability to exactly mimic the physiological environment of the body. To address this limitation, methods that can show in real time the binding interaction profile of heme and heme-DNA aptamers in physiological medium, such as surface plasmon resonance or newer technique like thermostable-Raman-interaction-profiling, need to be employed. Lastly, there is a need to examine the mode of interaction between heme and its DNA aptamer.

## List of abbreviations

EtOH, Ethanol; PBS, Phosphate buffer saline; PBST, Phosphate buffer saline tween 20; COL-TEG, Cholesterol triethylene glycol; >, Greater than; H_2_SO_4_, Sulphuric acid; *P. falciparum*, *Plasmodium falciparum*; ^∘^C, Degree Celsius; DMSO, Dimethyl sulfoxide; idT, Inverted deoxythymidine; μl, Microliter; RBC, Red blood cell; USA, United State of America, Hb, Hemoglobin; g, Gram; STD, Standard; <, Less than; μM, Micromolar; DNA, Deoxyribonucleic acid.

## CRediT authorship contribution statement

Abdul Wahab Aliyu: Performed the experiments; Analyzed and interpreted the data; Wrote the paper.

Muhammad Najmi Mohd Nazri: Performed the experiments.

Nur Fatihah Mohd Zaidi: Contributed reagents, materials, analysis tools or data.

Khairul Muhd Fadzli Mustaffa: Conceived and designed the experiments; Analyzed and interpreted the data; Contributed reagents, materials, analysis tools or data.

## Declaration of Competing Interest

The authors declare the following financial interests/personal relationships which may be considered as potential competing interests: Khairul Mohd Fadzli Mustaffa reports financial support was provided by 10.13039/501100015515Ministry of Education Malaysia.
